# Exploring the relationship between traffic, speed, and personal in-vehicle noise exposure during commutes

**DOI:** 10.1016/j.trip.2026.101992

**Published:** 2026-04-15

**Authors:** Abhay Anand, Shane J. Sacco, Misti Levy Zamora

**Affiliations:** UConn Health, Farmington, CT, United States

**Keywords:** Noise pollution, In-vehicle noise, Commute exposure, Driving speed, Traffic volume, Rush hour commute

## Abstract

While roadway noise is well-documented, research seldom quantifies the more critical in-vehicle exposure or evaluates the factors that modify it under real-world conditions. This study addresses this gap by investigating how traffic volume and driving speed, two key determinants in transportation planning, influence interior noise levels. Furthermore, we evaluate shifting commute times from rush hour to off-peak periods as a potential noise mitigation strategy.

We collected 1-minute noise and GPS data from 148 vehicle trips taken by 14 participants in Hartford County, US. To evaluate how the departure time of morning commutes to work impacts noise exposure, we compared noise levels during a rush-hour commute (6:30–8:30 am) to a non-rush-hour commute for each participant.

The median 1-minute noise level was 69.4 dBA (IQR = 65.7–74.1). Nine percent of measurements exceeded 80.0 dBA, the threshold of potential long-term hearing damage. Mixed-effects modeling showed both traffic volume and driving speed were positively associated with interior noise, with IQR increases linked to noise increases of 1.18 dBA and 0.71 dBA, respectively. However, substantial unexplained variability in the model suggests that other factors significantly influence noise levels more. The trip-averaged noise levels did not vary significantly between rush-hour (median = 70.7 dBA) and non-rush hour (median = 72.4 dBA) commutes (p = 0.84). This indicates that shifting commute times may not effectively mitigate overall noise exposure. Instead, strategies targeting overall traffic reduction may be more impactful.

By linking vehicle interior noise to traffic characteristics and travel behavior, this study provides an interdisciplinary basis for transportation policies and traffic management reducing the environmental health burdens of commuting.

## 1. Introduction

Noise associated with road traffic is pervasive and increasing, especially in urban areas (Wang et al., 2016; Brink et al., 2022). Road traffic noise has been associated with ischemic heart disease, hypertension, diabetes, annoyance, and sleep disturbance (Babisch, 2014; Clark et al., 2017; Guski et al., 2017; van Kempen and Babisch, 2012; Basner and McGuire, 2018).

Traffic noise can exhibit significant spatial and temporal variation within urban areas (Morelli et al., 2015; Wang et al., 2016). Morelli et al., 2015 found that the noise levels at locations near traffic were 7–12 dBA higher than at locations away from traffic in the European cities of Basel, Girona, and Grenoble. This variation is influenced by factors such as traffic volume, vehicle class mix, vehicle speed, and pavement type (Brink et al., 2022; [Bibr R34][Bibr R28]; Cook et al., 2023. However, most of this research focused only on ambient traffic noise (i. e., noise occurring on the exterior of the vehicle), and there is a scarcity of studies focusing on the influence of the same factors on vehicle *interior* noise, which is likely a better representation of true exposures to drivers and their passengers.

Noise inside a vehicle can originate from many sources. These include external factors like ambient road noise and interactions between the vehicle and its surroundings (e.g., tire and pavement interaction noise and wind noise), as well as internal factors like vehicle components such as air conditioning and horn use (Krishnamurthy and Hansen, 2014; Crocker, 2007). Few studies have investigated the association of vehicle interior noise with factors such as driving speed (Flor et al., 2020; Li et al., 2016b), pavement type (Li et al., 2016a), and mileage and production year of the vehicle (Przydatek et al., 2023). However, all these studies on vehicle interior noise have limited their measurements to a specific vehicle model and/or a select road section only. Krishnamurthy and Hansen, 2014 and Hansen (2002) noted that the noise inside the vehicle can vary significantly with the make of the vehicle and driving conditions. Hence, it is important to account for the variability of vehicle interior noise across vehicle makes and models as well as various real-world driving conditions in assessing the variability of vehicle interior noise.

Exposure to vehicle interior noise is particularly important in the U. S., where most people rely on their vehicles for transportation. In 2022, the average one-way travel time to work was 26.4 min and can be substantially longer (e.g., >1 h) for many (8.5%) Americans (U.S. Census Bureau, 2022). A peak period of commute to the workplace occurs between 6:00 am and 9:00 am, with 83.3% of U.S. workers traveling during this time (Burrows and Burd, 2024). Consequently, the peak roadway volumes of the commuters occur during these ‘rush hours’, which could lead to frequent stop-and-go traffic. More recently, many commuters are being offered flexible commuting opportunities. According to the U.S. Bureau Of Labor Statistics, 57% of salary workers had a flexible schedule in which they were able to vary the times they began and stopped working, and it has been suggested that more companies are going to continue to support more flexible work arrangements in the wake of the pandemic (U.S. Bureau of Labor Statistics, 2019; Kumar et al., 2023). With this, some commuters may have the choice of altering the departure time to work from heavy traffic rush hours to less-trafficked non-rush hours. In the context of noise pollution, a study conducted in the UK found that the ambient road traffic noise was up to 18% lower during rush hours as compared to non-rush hours, attributed to the higher driving speeds during non-rush hours (Ghaffarpasand et al., 2023). However, no studies have yet examined how changing morning commute times may affect exposure to vehicle *interior* noise.

To address this gap, this pilot study aimed to investigate initial associations and establish the feasibility of collecting real-world data on the influence of traffic volume and driving speed on vehicle interior noise across various vehicle models and driving conditions. Unlike traditional noise studies that rely on controlled settings, this research utilizes a participatory monitoring approach to assess the noise exposure commuters experience during their daily transit. Specifically, we investigated the relative importance of these factors among other uncontrolled variables utilizing real-time vehicle interior noise measurements, GPS logs, and publicly available information on annual average daily traffic. We also explored the potential of shifting commute times from morning rush hours to non-rush hours to modify in-vehicle noise exposure, providing valuable preliminary information for potential public health interventions and informing the design of future, larger-scale research.

## 2. Methodology

### 2.1. Study population

This study consisted of 14 participants who live or work in Hartford County, the second-most populous county in Connecticut, United States. Participants were a subset of the ongoing Effects of Transportation Choices on Commuter Health (ETCH) Study, which investigates whether modifying the morning departure time to work from rush hours (6:30 am to 8:30 am) to non-rush hours can influence personal exposure to pollution sources and their associated acute health impacts. The ETCH study recruited those individuals who met the criteria of having a regular commute longer than 20 min and traveling at least 5 miles on routes including heavily trafficked roads. Individuals who were under 18, pregnant, taking hypertension medications, or had a history of chronic lung disease or heavy tobacco smoke exposure were excluded. All participants provided informed consent, and the Institutional Review Board approved the study. This study was reviewed and approved by UConn Health Institutional Review Board with the approval number: 22–272–1, dated 06/21/2022.

The analysis presented in this manuscript is based on an initial phase of data collection, encompassing 14 participants. This early investigation was undertaken to primarily test the feasibility and robustness of our methodology for collecting highly time-resolved in-vehicle noise, GPS, and traffic volume data simultaneously across diverse real-world driving conditions and vehicle types. A pilot study of this scale allowed for the identification of logistical challenges and data collection nuances inherent in a participant-based field study.

### 2.2. Data collection Methods

Data was collected between February 2022 and July 2022. As per the ETCH study procedure, each of the 14 participants was asked to commute to work during morning rush hours for two consecutive days and outside the morning rush hours for another two consecutive days. The morning rush hour was defined as between 6:30 am and 8:30 am (U. S. Bureau of Labor Statistics, 2019). On the first day of each of the 2-day monitoring periods, a study member met with the participant and delivered a lightweight backpack containing instruments to monitor noise pollution, air pollution, and location. On the first day, each participant completed a demographic questionnaire (age, sex, health status, etc.), as well as a questionnaire asking about their home, work, and commute environments. On each monitoring day, the participant completed a time-activity diary questionnaire, which included commute-related information.

While the ETCH study protocol involved two-day monitoring periods for both the rush-hour and non-rush-hour conditions, the analysis presented in this article utilized data from the first day of each scenario only. Data from the second day were excluded because the 30-hour battery life of the GPS device resulted in incomplete location and speed information.

### 2.3. Exposure assessment

As part of the ETCH study protocol, participants were asked to carry the backpack with them as they conducted their typical daily activities. The backpack contained: (1) a noise monitoring instrument, TSI Quest Edge 4P noise dosimeter, (2) a GPS receiver, BT Q1000XT (Qstarz International, Taiwan), and (3) a custom multiple air pollution monitoring instrument (Buehler et al., 2022). Data were logged at 1-min resolution or higher for the noise dosimeter and GPS receiver.

For the rush hour/non-rush hour comparison, three participants were excluded as two did not travel along the same route during their rush hour and non-rush hour morning commutes, and one had incomplete information on location and driving speed during the morning commutes. However, these three participants were included in the linear mixed-effects model analysis assessing the influence of traffic volume and driving speed, as long as valid noise, speed and traffic volume data were available for their commutes. These exclusions, while impacting the analytical sample for specific analyses within this pilot, provided critical insights into potential limitations of the monitoring equipment and participant adherence in real-world settings. Such learnings are invaluable for refining protocols and optimizing data collection strategies for future large-scale research.

#### 2.3.1. Noise measurements

The TSI Quest Edge 4P noise dosimeters measured 1-minute averaged noise levels (Lavg) using a 5 dB exchange rate, and slow response. The noise estimates using a 5 dB exchange rate (specified by OSHA standard) can result in lower noise level estimates as compared to the more widely used 3 dB exchange rate (specified by NIOSH standard) (Jacobs et al., 2020). However, the noise estimates using both exchange rates were found to be highly correlated and using either exchange rate would not likely make any significant difference in the slope of the exposure–response relationship (Seixas et al., 2005). The measurements were A-weighted, a weighting method that corresponds to the sensitivity of the human ear. When noise exposure levels were below the lower detection limit of 60.0 dBA, values were replaced with 42.4 dBA, following recommendations of past studies (Hornung and Reed, 1990).

#### 2.3.2. Location and driving speed measurements

Participant location was tracked using BT-Q1000XT GPS receivers, allowing for accurate microenvironment identification. GPS receivers also provided real-time information on participant speed. Participants were assumed to be driving or riding in a vehicle if speeds were above 6.4 km/h (Zamora et al., 2018).

#### 2.3.3. Traffic volume

The Annual Average Daily Traffic (AADT) for the roads used by participants was obtained from the publicly available GIS dataset provided by the Connecticut Department of Transportation. AADT is widely used as a proxy for traffic volume when real-time traffic volume information is unavailable (Cook et al., 2023; Morley and Gulliver, 2016). In this study, AADT data were available for all major roads except low-traffic residential streets that provide access to residential areas from major roads. Minor roads that provide access to residential areas from major roads were excluded from analyses as AADT information was unavailable. QGIS 3.34.3 was used to obtain AADT values for each major road used by participants, matching the geocoded locations of commute routes. For easier interpretation, AADT is henceforth referred to as ‘traffic volume’ in the following sections.

### 2.4. Data Processing and Quality Control

As noted in Section 2.2, analysis was restricted to data from the first day of each scenario due to GPS battery limitations. A total of 148 vehicle trips were completed by participants, resulting in 2435 one-minute measurements on major roads. These trips include primary morning and afternoon work commutes as well as secondary trips such as midday errands and non-work-related activities. Of these, 350 min were missing noise values, and 29 min were missing speed values.

For the analysis comparing rush hour and non-rush hour morning commutes, three participants were further excluded: two did not travel along the same route, and one had incomplete information on location and driving speed during the morning commutes. However, these three participants were included in the linear mixed-effects model analysis assessing the influence of traffic volume and driving speed, as long as valid noise, speed and traffic volume data were available for their commutes. These exclusions, while impacting the analytical sample for specific analyses within this pilot, provided critical insights into potential limitations of the monitoring equipment and participant adherence in real-world settings, invaluable for refining protocols for future research.

### 2.5. Data analysis

To compare continuous variables by commute time (rush vs. non-rush hour), we conducted Wilcoxon signed rank tests within participants and Mann-Whitney U tests between participants. To determine associations between continuous variables, we calculated Spearman rank correlations.

To estimate the association of noise levels with driving speed and traffic volume, a linear mixed-effects model was created using the lme4 package (Bates et al., 2015) in R (R Core Team, 2024)). One-minute noise levels were the dependent variable, while driving speed and traffic volume were fixed effects, and participant and trip IDs were included as nested random effects. A mixed-effects model was chosen because the data are nested (1-minute observations within trips, clustered within participants). Observations within the same vehicle or trip are likely correlated due to shared characteristics. By assigning each participant and trip a unique random intercept, this mixed-effects modeling approach effectively controls for unmeasured vehicle-specific and trip-specific variables. Associations are reported as increases in noise levels (dBA) per Interquartile Range (IQR) increase in the driving speed and traffic volume respectively. The IQR (defined as the difference between 25th and 75th percentiles) was utilized to reflect the noise impact of moving from a ‘typical low’ to a ‘typical high’ value for driving speed and traffic volume within our specific dataset, thereby providing a more practically meaningful estimate than a mathematically small 1-unit increases in those variables.

All analyses were conducted in R version 4.3.3 (R Core Team, 2024). Alpha for two-sided tests was set to 0.05.

## 3. Results and discussion

### 3.1. Participant characteristics

Participant characteristics are summarized in Table 1. There were 10 female and 4 male participants, with ages ranging from 22 to 64 years (median: 24.5 years; IQR = 24.0–31.2). The study successfully captured a diverse fleet of vehicles, a strength for assessing real-world exposure variability. Vehicles included six sport utility vehicles (SUVs), five sedans, two hatchbacks, and one coupe. The wide range of vehicle ages, from 1 to 19 years (median: 9.5 years; IQR = 4.2–12.7), and engine power, from 122 to 350 horsepower (median: 177 horsepower; IQR = 158–254), suggests that noise measurements were collected across vehicles with highly variable acoustic properties.

There was a significant operational variation observed in window usage, with 43% of participants reported that they regularly drove their vehicles with the windows in both open and closed positions and an equal 43% reporting always closing them. As opening the window introduces the wind noise, those participants who vary their window use could see significant variability in noise between trips.

### 3.2. Variation of vehicle interior noise during all types of trips during a day

In total, 148 trips were completed by the 14 participants, resulting in 2,435 one-minute noise measurements on major roads. The trips occurred throughout the day, with 35% of the trips occurring during the morning (5 am-12 pm), 54% during the afternoon (12 pm- 7 pm), and 11% during the late evening (7 pm- 11 pm). The median duration of trips was 13 min (IQR = 7–20). These trips captured a wide range of operational conditions, with a median driving speed of 59.4 km/h (IQR = 43.7–90.6) and a median traffic volume of 17,200 vehicles/day (IQR = 10,000–30,350). The wide variability in these speed and traffic volume measurements demonstrates that the study successfully characterized a highly heterogeneous driving environment, encompassing conditions from slower-moving traffic to free-flow traffic.

**Fig. 1.**
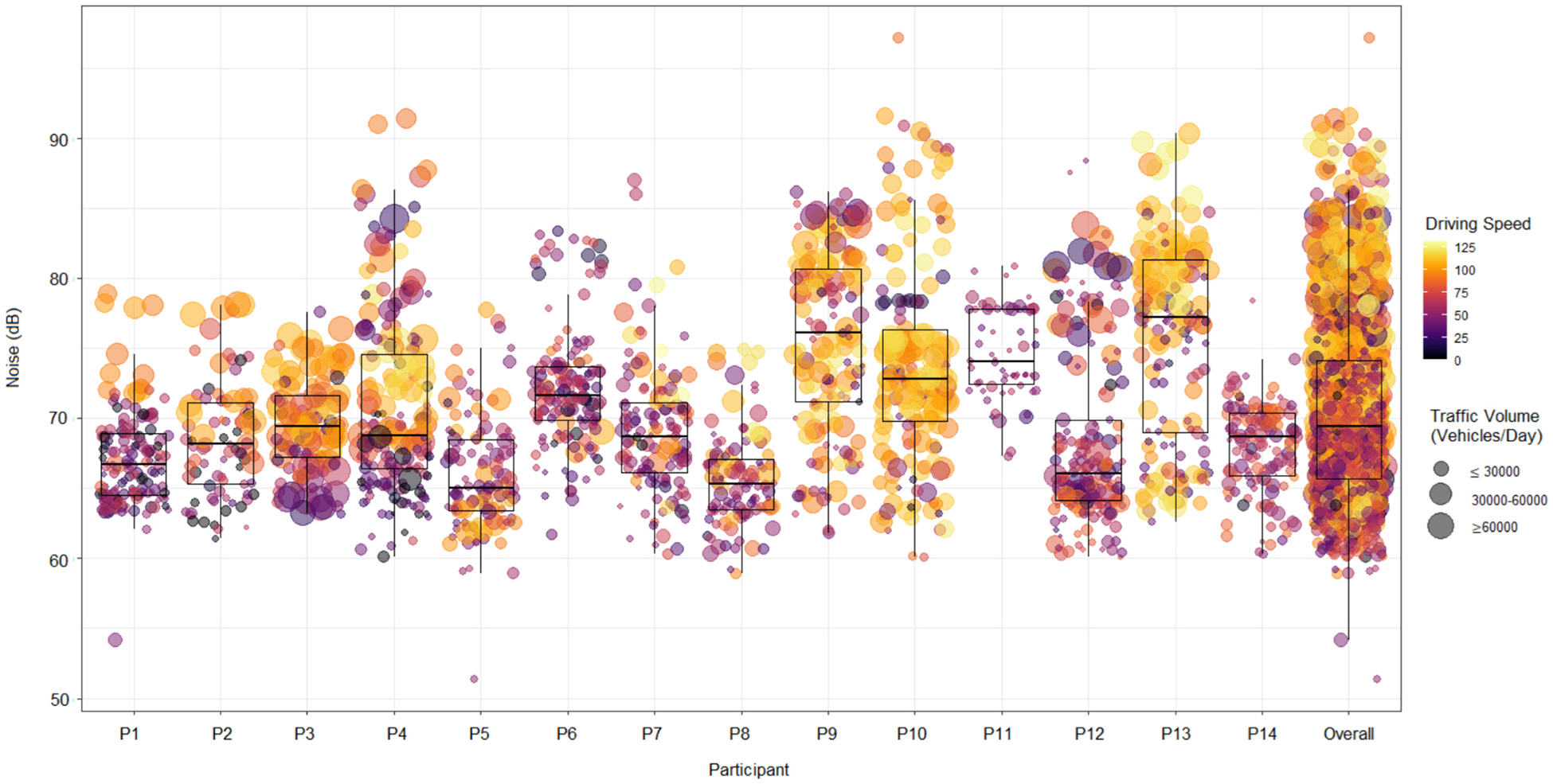
Distribution of 1-minute averaged noise levels grouped by driving speed and traffic volume on the roads during commute for each of the 14 participants.

Fig. 1 shows the distribution of 1-minute noise levels for each participant, representing all daily trips captured in this study. The overall median 1-minute noise level across all measurements was 69.4 dBA (IQR = 65.7–74.1). More importantly, of all the 1-minute noise level measurements, nine percent were above 80.0 dBA, which is the threshold level that OSHA considers a risk for long-term hearing damage.

The distribution of noise levels, summarized in Fig. 1, also shows the significant variation between participants. The median noise levels for individual participants varied widely, ranging from 65.0 dBA to 77.2 dBA. These between-participant differences could be partly due to fixed factors related to the vehicle, such as its manufacturer, model, engine power, and age, that can create distinct baseline noise characteristics.

**Fig. 2.**
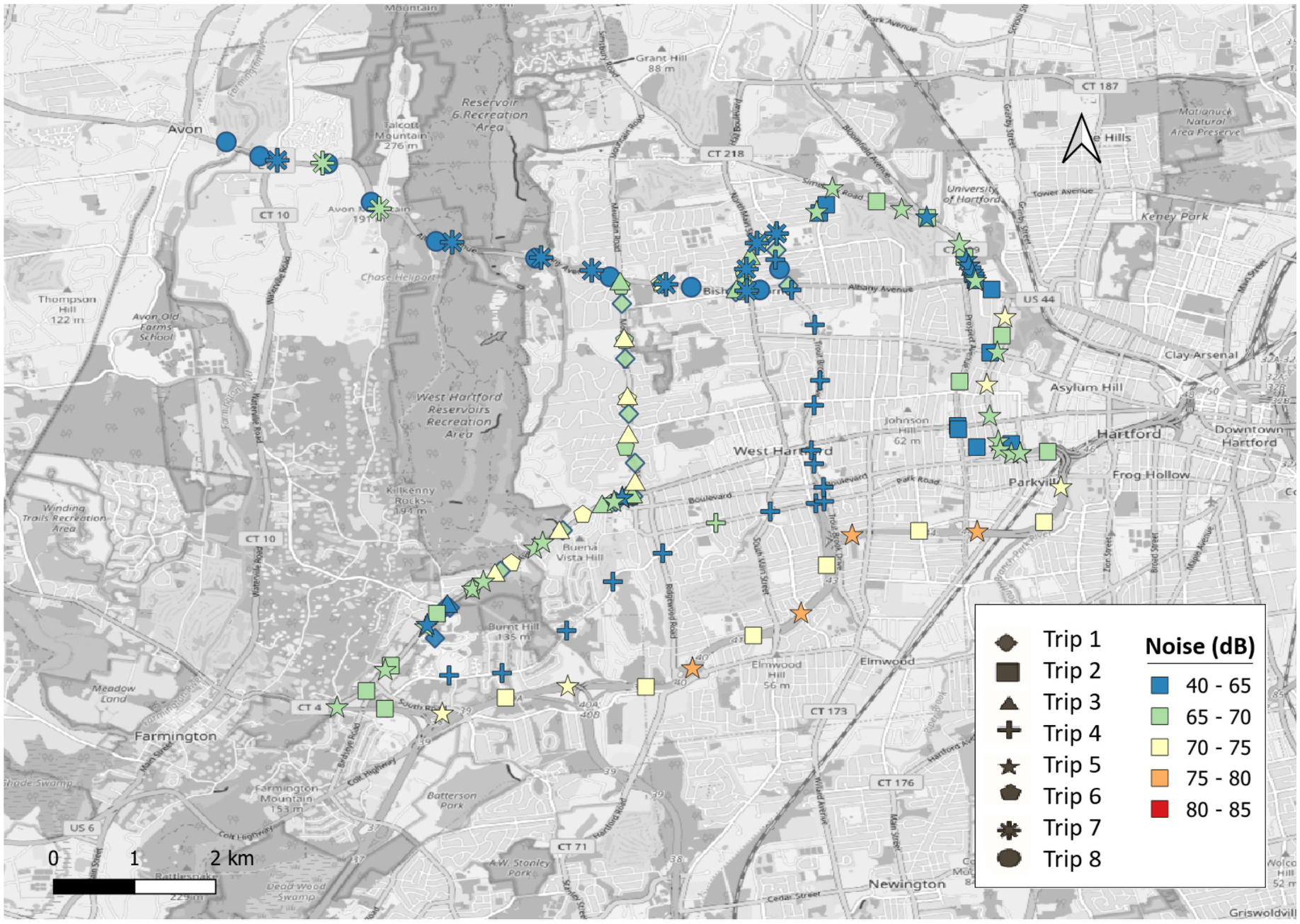
Distribution of 1-minute noise levels for participant P1 across all trips.

Apart from this between-participant variability, there was also a significant variability within each participant’s noise exposure. This is illustrated in Fig. 2, which shows the noise levels for participant P1 across all the trips. It can be seen that the noise levels varied significantly between the trips. Moreover, the noise levels also varied within the same individual trip.

The between-trip variation of noise levels within each participant is illustrated in detail in Fig. S1. It can be seen that certain trips such as trip 4 of participant P4 and trip 6 of participant P6 were distinctively higher compared to other trips of the respective participants. These large between-trip differences for individual participants could be mainly due to trip-specific operational factors, such as the use of air conditioning, whether windows were up or down, and music volume.

The high degree of minute-by-minute change in noise levels observed within each trip (see Fig. 2) is largely driven by factors that change significantly during a trip, including driving speed and traffic volume. Fig. 1 shows a visual preview of the influence of driving speed and traffic volume on the noise levels, as the individual data points are categorized by driving speed and traffic volume. In the following section, we quantify that relationship via statistical modeling.

### 3.3. Influence of driving speed and traffic volume on vehicle interior noise

The significant minute-by-minute variability observed within trips (discussed in Section 3.2) indicates that dynamic factors, such as driving speed and traffic volume, significantly influence real-time noise exposure. A linear mixed-effects model was therefore employed to quantify the association of driving speed and traffic volume with noise levels. The model assessed the specific influence of these factors, after accurately accounting for the significant unmeasured variability observed between different trips and individual drivers (by including trip and participant as random effects). The results are presented in Table 2.

Driving speed and traffic volume were positively associated with noise levels (ps < 0.001). Specifically, an increase of 47 km/h in driving speed (representing the IQR for driving speed in this study) was associated with a 1.18 dB increase in noise (95% CI: 0.85–1.46). Similarly, an increase of 20,350 vehicles/day in traffic volume (representing the IQR for traffic volume in this study) was associated with a 0.71 dB increase in noise (95% CI: 0.51–0.92). By reporting these associations per IQR, the results reflect the noise impact of moving from a ‘typical low’ to a typical ‘high’ value for driving speed and traffic volume in our study area. These findings validate the limited previous research that linked driving speed to vehicle interior noise in single-vehicle studies (Flor et al., 2020; Li et al., 2016b), and confirm associations widely established for ambient noise (Morelli et al., 2015; Brink et al., 2022; Wang et al., 2016). These results show the direction and magnitude of associations within this pilot group of participants (N = 14), providing a basis for future larger-scale research.

However, combined driving speed and traffic volume only accounted for a small 4.5% of the total variance in interior noise levels (marginal R^2^ = 0.045). The total variability explained by the overall model was much higher, at 73% (conditional R^2^ = 0.73). This shows that the majority of the explained variability in noise levels is captured by the unmeasured differences between individual trips and participants, rather than by the direct influence of speed or traffic volume.

Partitioning the total noise variability between the participant and trip levels determined which grouping factor contributed most to the explained variation. The results showed that unaccounted factors specific to individual trips were the overwhelmingly dominant influence on noise levels. The variability attributable to individual trips accounted for 55% of the total noise variance (Trip-level Variance Partition Coefficient, VPC: 0.55). This proportion was significantly higher than the 17% of variance attributed to the participant’s fixed characteristics (Participant-level Intraclass Correlation Coefficient, ICC: 0.17). This difference indicates that factors that change from trip to trip—rather than the inherent traits of the driver or vehicle—play the dominant role in influencing overall noise variability.

These trip-specific conditions include factors like air conditioning, windows being up or down, music volume, type of road surface, and weather conditions; all of which can change significantly from one trip to another (Soeta and Sakamoto, 2018; Prasad et al., 2009; Krishnamurthy and Hansen, 2014; Li et al., 2016b; Ergun et al., 2024). Krishnamurthy and Hansen (2014) observed that during most vehicle trips, a single noise source tends to be the most dominant, effectively masking the influence of other noise sources present during the trip. For example, with the air conditioning switched on and windows closed, air conditioning was the dominant source of noise inside the vehicle, masking road noise and engine noise (Krishnamurthy and Hansen, 2014). Similarly, with air conditioning switched off and windows opened, wind noise became the dominant source, masking road noise and engine noise. Therefore, the distinct prominence of individual noise components leads to substantial variations in overall noise characteristics across different vehicle trips, highlighting the necessity of data acquisition across diverse operational conditions that include all combinations of potential noise sources.

### 3.4. Effect of modifying the departure time to work on vehicle interior noise levels

**Fig. 3.**
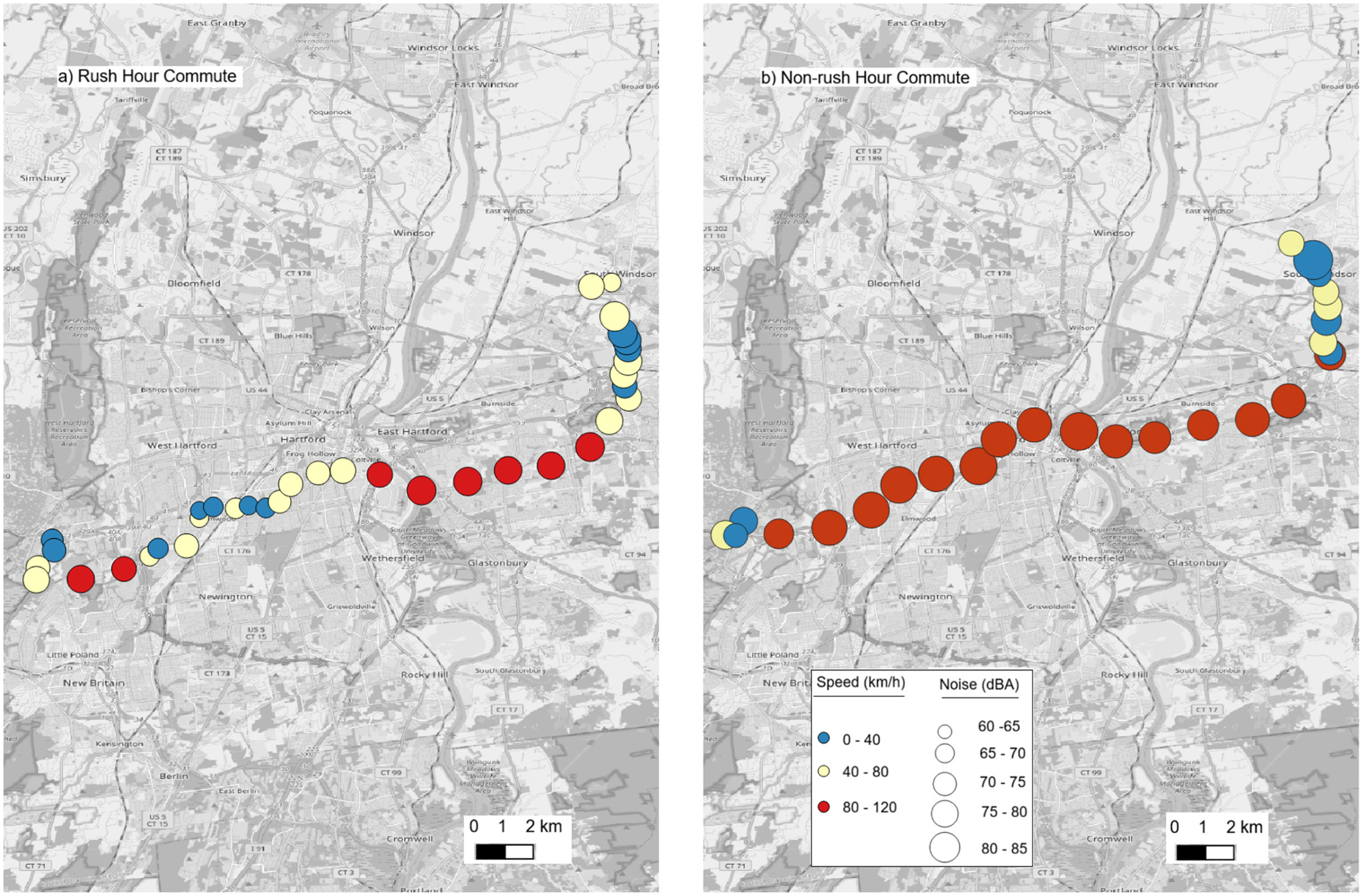
Spatial and temporal variability of vehicle interior noise and driving speed during a) rush hour commute and b) non-rush hour commute for participant P3.

The preceding Sections 3.1–3.3 of this study examined all trips collected from the participants. In contrast, the analysis presented in this section focuses exclusively on the participants’ morning commute trips to work. We investigated whether changing the departure time to work from morning rush hours to non-rush hours can modify exposure to vehicle interior noise. Fig. 3 shows a detailed spatial illustration of minute-by-minute noise variability along the route for one of the participants during the rush hour and non-rush hour commute periods.

Participants reported significant differences in traffic conditions between the periods, with 73% reporting moderate or heavy traffic during rush hour commutes compared to only 18% during non-rush hours. However, despite these perceived differences, there was no statistically significant difference in either driving speed or trip duration between the two commute periods. The median trip-averaged driving speed was 58.0 km/h (IQR = 53.0–65.5) for rush hour commutes, which was not significantly different from that for non-rush hour commutes of 63.5 km/h (IQR = 54.4–71.1) (p = 0.65). Similarly, the median trip duration for rush hour commutes (25 min, IQR = 20–31) did not significantly differ from the non-rush hour duration (26 min, IQR = 19–27) (p = 0.84).

**Fig. 4.**
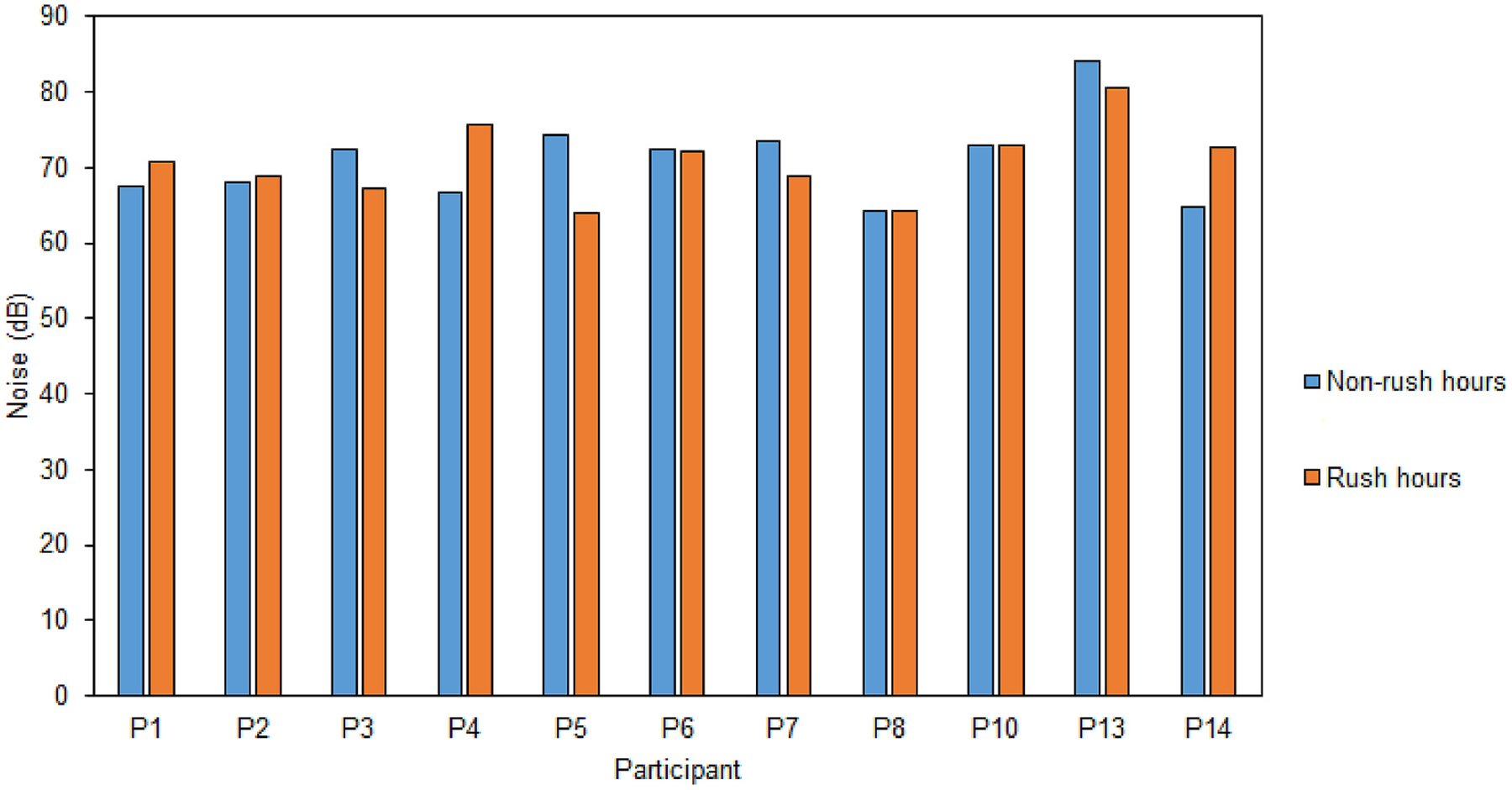
Comparison of Trip-averaged Noise levels between the two commute periods for each participant.

Similar to the driving speed and trip duration, the trip-averaged noise levels also did not statistically differ between the rush hour (median: 70.7 dBA; IQR = 68.0–72.8) and non-rush hour (median: 72.4 dBA; IQR = 67.1–73.2) commute periods (p = 0.84) (Fig. 4). The exact reasons for this non-significant difference in noise levels remain inconclusive because, unlike speed, we lacked trip-specific information for other key influencing factors, such as trip-specific traffic volume data.

However, a detailed participant-level analysis showed that only two of the participants (P3 and P7) experienced significant differences in traffic conditions (as inferred by differences in speed and duration) between the commute periods. Both participants (P3 and P7) had significantly lower noise levels during rush hour commutes (Fig. 3 and Fig. S2). These rush hour trips were characterized by longer travel times (at least 5 min; see Fig. S3) and slower driving speeds (p < 0.10; see Fig. S4). Furthermore, both participants exhibited a moderate-to-strong correlation between 1-minute noise levels and driving speed or traffic volume (*ρ* ≥ 0.40; *p* ≤ 0.10). This finding aligns with the findings of Li et al., 2016a that higher driving speeds on an arterial road under free-flow conditions resulted in increased interior vehicle noise compared to congested traffic on the same road. However, the generalizability of this finding in our study is limited by the small sample size of two participants, necessitating further investigation with a larger sample.

### 3.5. Implications

This study revealed that a considerable proportion of the vehicle interior noise measurements exceeded the OSHA threshold level associated with the potential for long-term hearing impairment. This could be more concerning for individuals with pre-existing auditory disorders, as well as for older adults, who exhibit increased vulnerability to noise-induced hearing loss. In addition to hearing impairment, chronic exposure to high vehicle interior noise levels can contribute to non-auditory health effects such as heart disease, hypertension, and insomnia that can occur at levels well below the threshold for hearing impairment (Eichwald et al., 2022; Neitzel et al., 2012). These findings underscore the need for future research to fully characterize the health impacts of long-term commute noise exposure, particularly in a car-dependent urban setting wherein the population spends a significant amount of time commuting each week.

This study also found that driving speed and traffic volume increase the vehicle interior noise levels. Thus, urban planners should consider incorporating noise mitigation measures into road design. However, results also show that additional unmeasured factors in this study may play a more significant role in determining vehicle interior noise levels, highlighting the need for further research in this area. While vehicle-specific factors can be easily identified and accounted for, collecting data on factors that vary throughout individual trips, particularly in large-scale versions of participatory studies like ours, may present significant logistical challenges, such as the risk of high participant burden from constant detailed logging and the possibility of inaccurate or biased self-reported trip details. This pilot study provided crucial preliminary insights into these logistical challenges, which are invaluable for optimizing data collection strategies and participant engagement in broader, future research efforts.

Additionally, given our findings, future research should concentrate on investigating the influence of morning commute time on noise levels along routes characterized by pronounced fluctuations in traffic conditions between morning rush hours and non-rush hours.

### 3.6. Strengths and limitations

This study has several strengths. We utilized simultaneous measurements of highly time-resolved vehicle interior noise, location, and driving speed across a large urban area. This provided a more spatially- and temporally-resolved understanding of vehicle interior noise exposures. Further, we assessed measurements in multiple vehicle models in real driving scenarios, helping provide a better understanding of true vehicle interior noise exposures and their variability. These methodological strengths are particularly valuable for a pilot study, as they demonstrate the feasibility and effectiveness of our approach in capturing complex, real-world exposures, laying a robust foundation for more extensive future investigations. Furthermore, by integrating high-resolution vehicle interior noise data with traffic characteristics and travel behavior, this study provides an interdisciplinary assessment of how transportation planning influences the personal noise exposures of the commuter.

The study has several limitations. Rush hour and non-rush hour commute noise were each measured only once per participant, on separate days. Thus, the daily variations in noise levels during each of the commute periods were not accounted for. Information on various factors, such as the opening of windows and sound system usage, which can influence vehicle interior noise, was unavailable. In the mixed-effect model analysis investigating the influence of driving speed and traffic volume on noise, we attempted to account for these factors by assigning random effects for each trip and participant. However, the influence of these unaccounted sources could not be fully adjusted for in analyses without collecting this data directly. These identified limitations are important insights gained from this pilot phase, highlighting areas for refinement and more detailed data collection strategies in subsequent, broader research efforts. While limiting the definitive conclusions of this initial study, they are crucial for guiding the design of future comprehensive investigations.

## 4. Conclusion

To our knowledge, this is the first study to investigate the influence of driving speed and traffic volume on vehicle interior noise across various vehicles and real-world driving conditions with highly resolved measurements. Our study assessed vehicle interior noise in 14 vehicles, with 148 trips along 191 distinct major roads. This approach, spanning multiple vehicle models and varied driving conditions, provided a valuable preliminary understanding of actual in-vehicle noise exposure and its inherent variability, contributing significantly to a field where such detailed, real-world data is scarce.

Elevated levels of vehicle interior noise were recorded in this study, suggesting a potential risk of auditory and non-auditory health effects from chronic exposure. In a car-dependent urban setting, with the population spending significant hours a week commuting, this highlights a potential public health issue that warrants further investigation. This initial investigation also successfully demonstrated the feasibility and value of collecting highly time-resolved, real-world in-vehicle noise exposure data across diverse commuting scenarios and multiple vehicle types, providing essential preliminary insights into a poorly characterized public health exposure.

Driving speed and traffic volume were found to increase vehicle interior noise levels, yet the results suggest that additional unmeasured factors in this study, especially specific to individual trips, may play a more significant role in determining these noise levels, highlighting the need for further research in this area.

Morning rush hour conditions did not result in significantly elevated vehicle interior noise levels relative to non-rush hour periods. Moreover, our findings hinted that increased speeds during non-rush hour times could potentially elevate noise levels, but were limited by sample size and warrant further research to validate this observation. These foundational findings from our pilot study underscore the public health relevance of in-vehicle noise exposure and provide critical methodological and substantive guidance for the design and execution of larger, more comprehensive studies aimed at informing effective interventions for traffic noise management.

## Supplementary Material

1

## Figures and Tables

**Table 1:** Participant characteristics.

	N
**Race**	
White	11
Asian	2
Prefer not to answer	1
**Gender**	
Male	4
Female	10
**Windows up or down while driving**	
Down	2
Up	6
Both	6
**Age of vehicle (years)** (mean ± sd)	9.1 ± 5.6
**Engine power of vehicle (hp)** (mean ± sd)	205 ± 68
**Manufacturer Suggested Retail Price of the vehicle (USD)** (mean ± sd)	22483 ± 4406

**Table 2:** Association of 1-minute vehicle interior noise levels with AADT on the road and driving speed (N = 2025). Associations were estimated using a mixed effects model with nested random intercepts (trip within participant).

	Estimates	95% CI	p-value
**AADT (IQR increase)**	0.71	0.51 – 0.92	<0.001
**Driving Speed (IQR increase)**	1.18	0.85 – 1.46	<0.001

## Data Availability

Data will be available from the corresponding author upon request.

## Appendix A. Supplementary data

Supplementary data to this article can be found online at https://doi.org/10.1016/j.trip.2026.101992.
