# Supplementary material for: Exploring the relationship between traffic, speed, and personal in-vehicle noise exposure during commutes
Source: Transp Res Interdiscip Perspect. Author manuscript; Available in PMC 2026 May 29. (PMC13218673; doi:10.1016/j.trip.2026.101992)
Supplement: 1 [file NIHMS2177738-supplement-1.docx]

**Supporting information for**

**Exploring the Relationship Between Traffic, Speed, and Personal In-Vehicle Noise Exposure During Commutes: A Pilot Study**


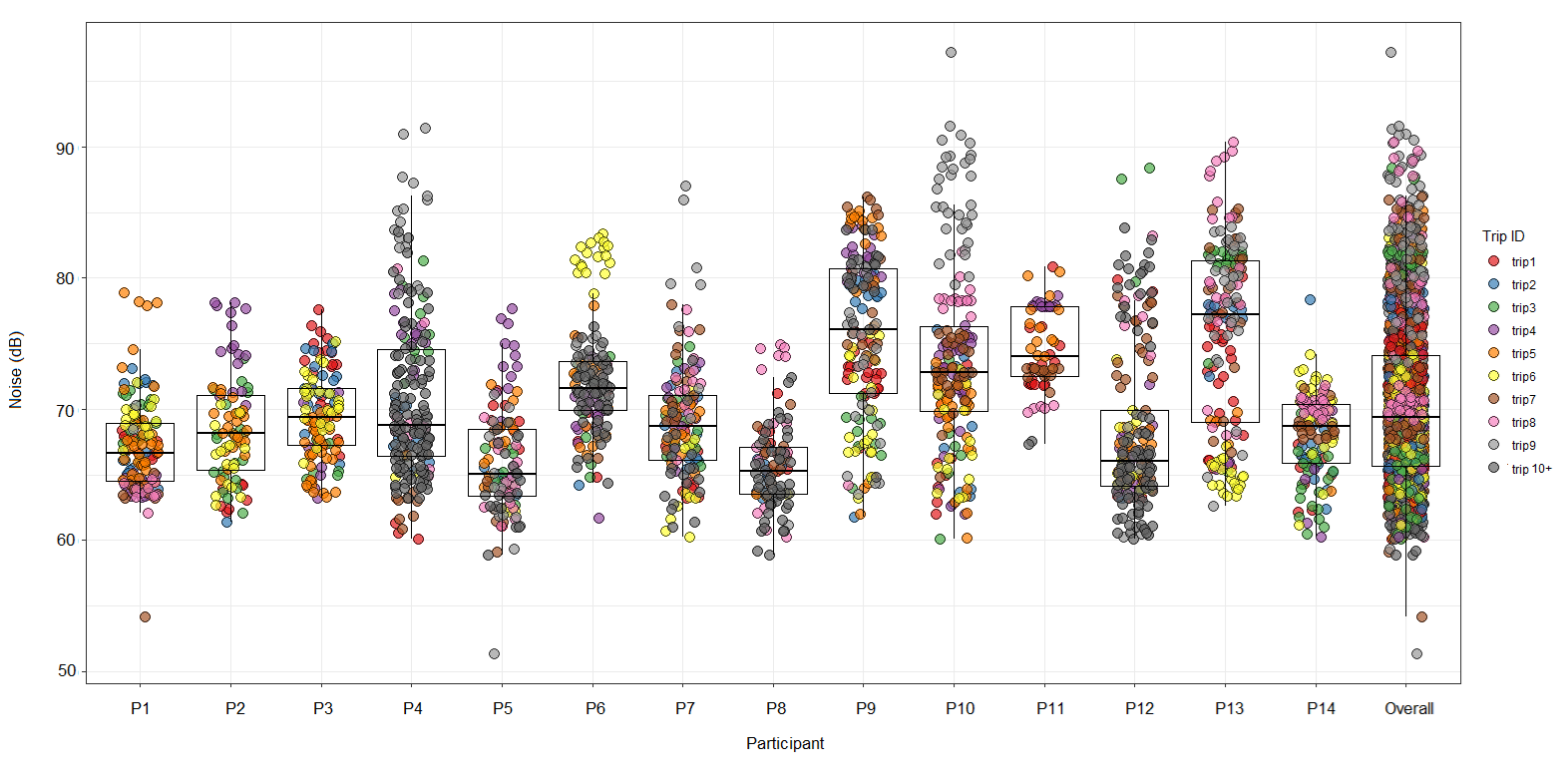


**Figure S1:** Distribution of 1-minute averaged noise levels grouped by trips for each of the 14 participants.


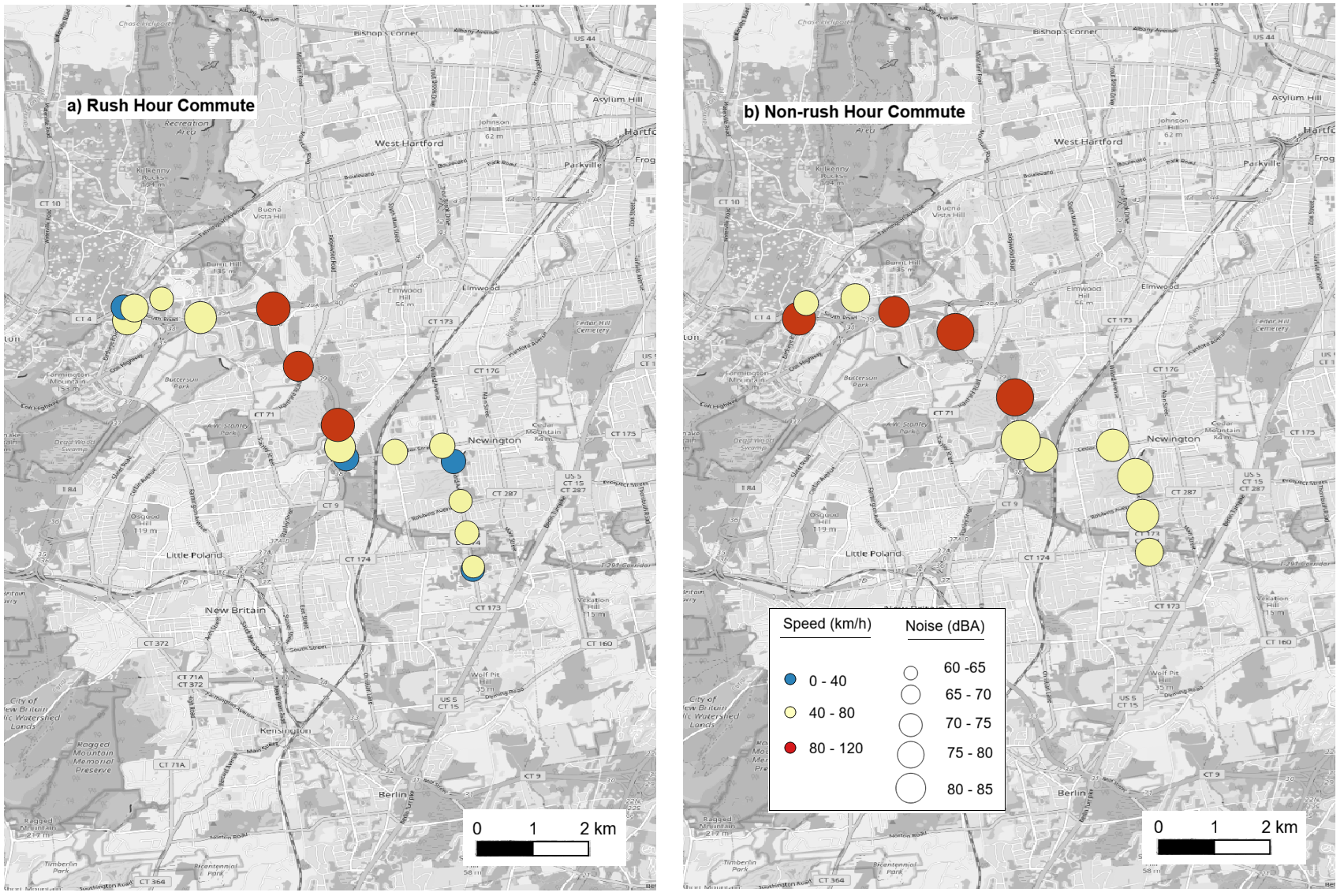


**Figure S2:** Spatial and temporal variability of vehicle interior noise and driving speed during a) rush hour commute and b) non-rush hour commute for participant P7.


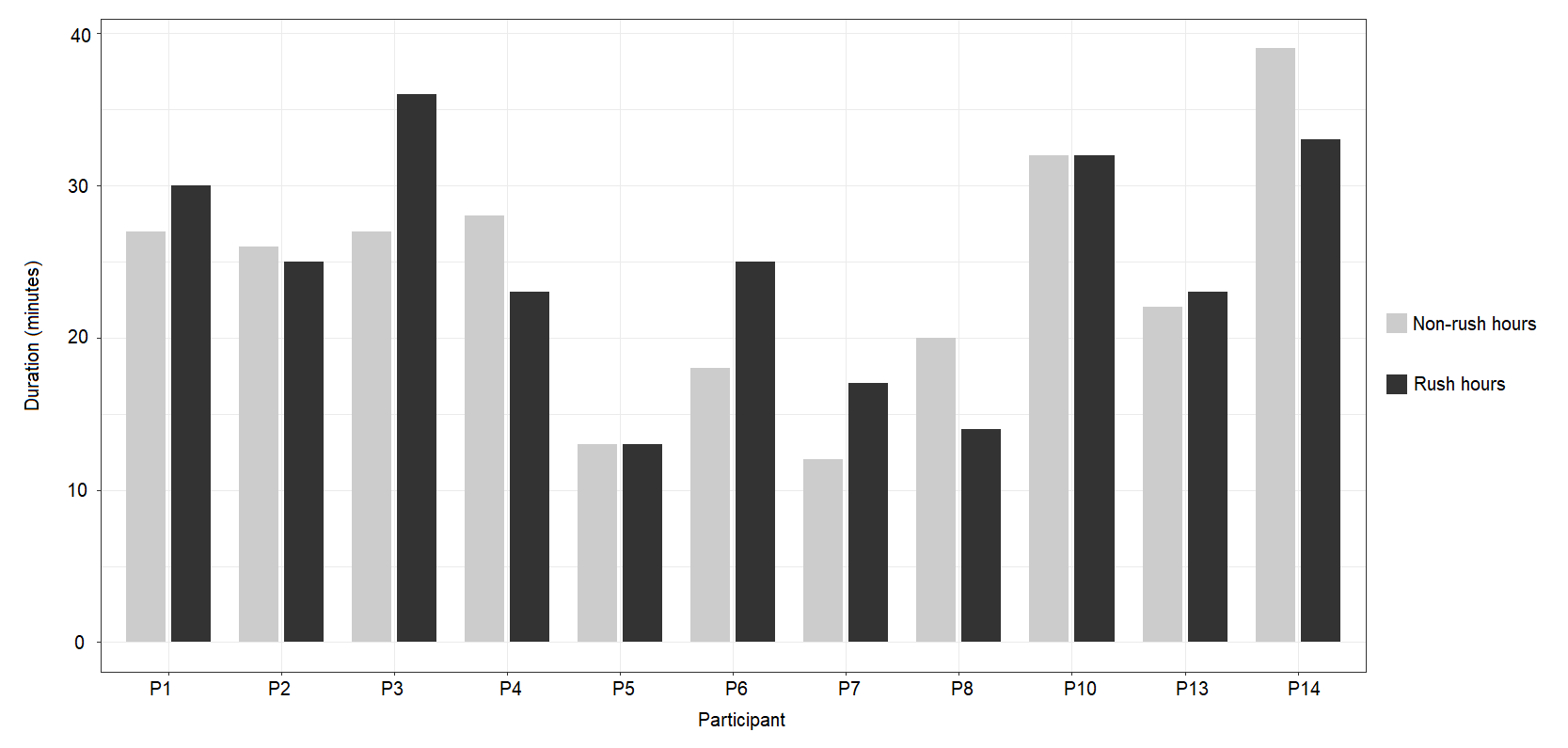


**Figure S3:** Comparison of the duration of the commute during rush hours and non-rush hours of the day for 11 participants.

**
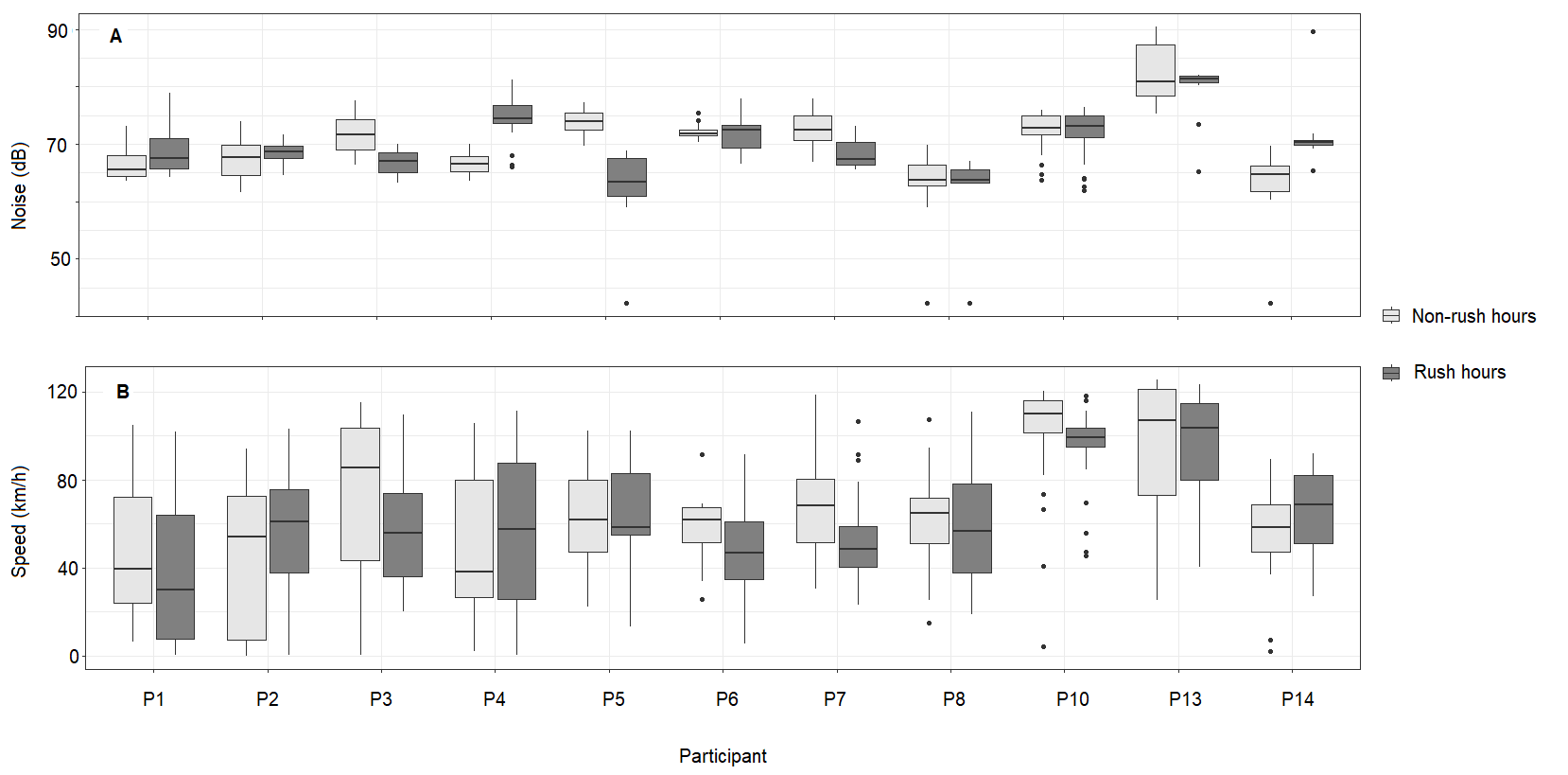
**

**Figure S4:** Comparison of the distributions of a) 1-minute averaged noise levels and b) 1-minute averaged driving speeds during the rush hour commutes and non-rush hour commutes of the day for 11 participants.

**Table S1:** Association of 1-minute vehicle interior noise levels with AADT on the road and driving speed after imputing missing Noise and driving speed data with respective median values (N=2025). Associations were estimated using a mixed effects model with nested random intercepts (trip within participant).

|  | **Estimates** | **95% CI** | **p-value** |
| --- | --- | --- | --- |
| **AADT (IQR increase)** | 0.75 | 0.55 – 0.95 | <0.001 |
| **Driving Speed (IQR increase)** | 1.22 | 0.92 – 1.51 | <0.001 |
